# Comparative genomics and evolutionary analysis of plant CNGCs

**DOI:** 10.1093/biomethods/bpac018

**Published:** 2022-08-17

**Authors:** Akram Ali Baloch, Kaleem U Kakar, Zarqa Nawaz, Muhammad Mushtaq, Asma Abro, Samiullah Khan, Abdul Latif

**Affiliations:** Department of Biotechnology, Faculty of Life Sciences, Balochistan University of Information Technology, Engineering and Management Sciences (BUITEMS), Quetta, Pakistan; Department of Microbiology, Faculty of Life Sciences, Balochistan University of Information Technology, Engineering and Management Sciences (BUITEMS), Quetta, Pakistan; Department of Botany, University of Central Punjab, Rawalpindi, Pakistan; Department of Biotechnology, Faculty of Life Sciences, Balochistan University of Information Technology, Engineering and Management Sciences (BUITEMS), Quetta, Pakistan; Department of Biotechnology, Faculty of Life Sciences, Balochistan University of Information Technology, Engineering and Management Sciences (BUITEMS), Quetta, Pakistan; Department of Biotechnology, Faculty of Life Sciences, Balochistan University of Information Technology, Engineering and Management Sciences (BUITEMS), Quetta, Pakistan; Department of Microbiology, Faculty of Life Sciences, Balochistan University of Information Technology, Engineering and Management Sciences (BUITEMS), Quetta, Pakistan

**Keywords:** CNGCs, phylogenetic analysis, evolution, duplication, synteny

## Abstract

Comparative genomics and computational biology offer powerful research tools for studying evolutionary mechanisms of organisms, and the identification and characterization of conserved/distant genes and gene families. The plant *CNGC* gene family encodes evolutionary conserved ion channel proteins involved in important signaling pathways and biological functions. The fundamental ideas and standard procedures for genome-wide identification and evolutionary analysis of plant cyclic nucleotide-gated ion channels employing various software, tools, and online servers have been discussed. In particular, this developed method focused on practical procedures involving the comparative analysis of paralogs and orthologs of *CNGC* genes in different plant species at different levels including phylogenetic analysis, nomenclature and classification, gene structure, molecular protein evolution, and duplication events as mechanisms of gene family expansion and synteny.

## Introduction

A gene family is a collection of multiple related genes that are similar in sequence (i.e. >50% pairwise amino acid similarity), structures, and biological functions. Of all the genes in sequenced eukaryotic and prokaryotic genomes, majority of these genes belong to one or other gene family. Cyclic nucleotide-gated ion channels, abbreviated as CNGCs, is one such family of evolutionarily conserved group of proteins that occur in all taxa of animals, plants [1, 2], and some prokaryotes [2], playing important biological functions [3]. These CNGC family proteins are mostly found in the plasma membrane [4, 5], vacuole membrane [6], or nuclear envelope of the eukaryotic cell [7], and perform multiple biological functions including the uptake of both essential and toxic cations, calcium signaling, growth and stress tolerance in plants [2, 4, 8–10], and essential for vision and olfaction in animals [11, 12].

CNGCs were initially studied in animal and *Arabidopsis* systems, but the advent of latest advanced sequencing and genomic techniques has led to the identification and characterization of CNGC family in many important crop genomes such as rice (*Oryza sativa* L.), tomato (*Solanum lycopersicum*), cabbage (*Brassica oleracea*), tobacco (*Nicotiana tabacum*), pear (*Pyrus bretchneideri* Rehd.), maize (*Zea mays*), and Rosaceae [3, 13–18].

These studies involving structural, functional, and evolutionary analysis of plant CNGCs have provided valuable information of their structural modules, underlying regulatory mechanisms and phylogenetic relationships with other channels. Similar to living organisms, the hierarchy of genes in a gene family imitates an ancient and ongoing evolutionary process [19, 20].

Therefore, studying the *CNGC* gene family is not only crucial for understanding its origin, evolution and gene and protein functions in plants, but this topic has become one of the most researched theme in comparative genomics and proteomics.

Several conceptual methods and analytical tools can and should be used for assessing homology and divergence, duplication events, phylogenetic relationships among genes, and reconstructing evolutionary events [20, 21].

A comprehensive identification of the *CNGC* genes in newly sequenced genomes, followed by authentic classification is a prerequisite for almost all sorts of interpretations about the evolution of *CNGC* genes and their encoded proteins. Extensive phylogenetic analysis can be useful to document *CNGC* gene family history, justify its nomenclature and classification, and fully understand the diversity and relatedness of individual members, groups, and species. Determining syntenic relationships between plant genomes based on colinear blocks provides valuable information about the evolutionary history of *CNGC* gene family, and paleopolyploidy and gene duplication events. Comparison of the exon–intron structures of individual *CNGC* genes is an important part of gene families’ evolutionary studies, which provides valuable information regarding the possible mechanisms of structural evolution of *CNGC* paralogs and additional proof of phylogenetic clustering [22]. According to evolutionary biologists, the protein molecular evolution is affected by both amino acid composition and functional requirements or selective constraints while the degree of effect of each factor (amino acid composition and functional constraints) varies. It is established that functionally important parts of protein molecule undergo gradual change during the process of evolution [23]. Therefore, comparative analysis of amino acid composition, physicochemical properties and motif composition of CNGC family proteins are not only important for functional characterization, but also helps in studying the molecular evolution of the CNGC family of different plant species and other proteins. The present protocol documents a step-by-step procedure and the use of different methods and techniques in comparative genomics and evolutionary analysis of the *CNGC* gene family in plants.

## Materials and methods

### Data mining and identification of plant *CNGC* gene family

**Figure 1:**
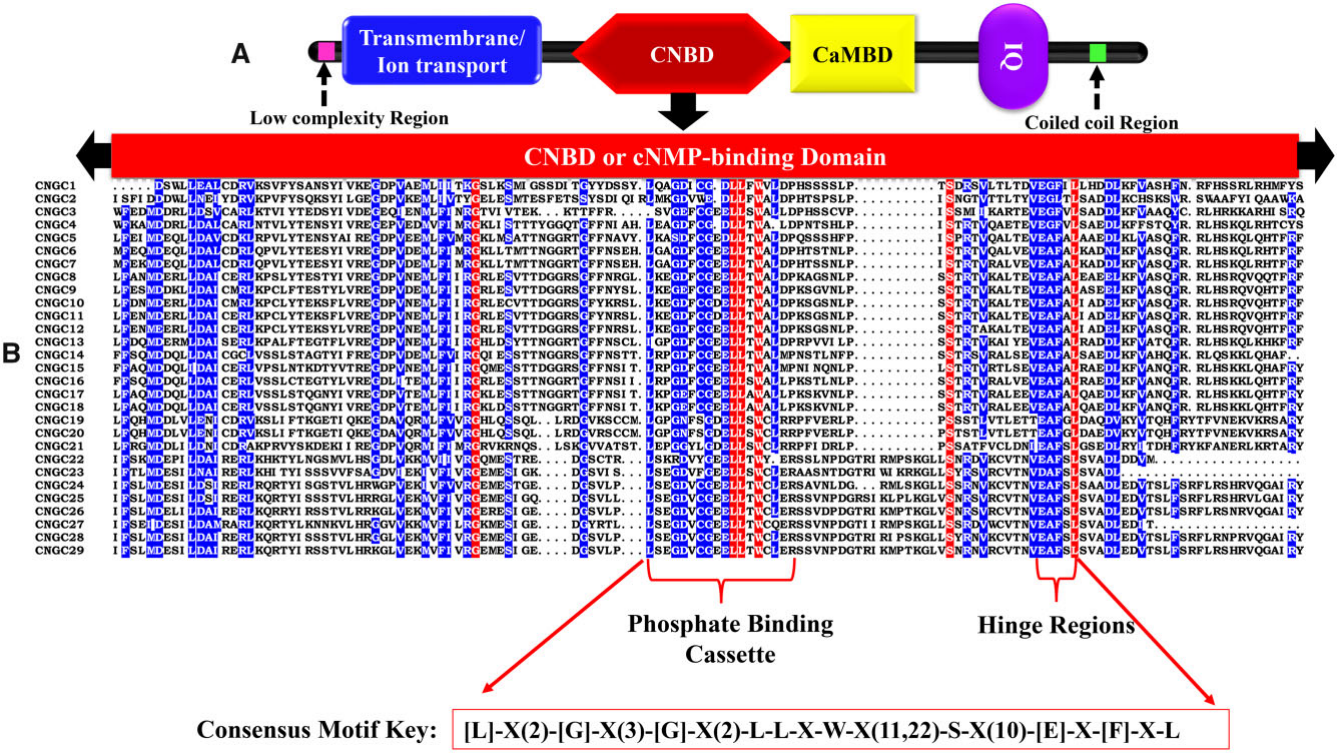
A cartoon model showing the characteristic domain architecture of plant CNGCs (a) and plant CNGC-specific consensus motif key showing conserved regions of CNBD spanning PBD and hinge regions (b). Amino acids allowed in a specific position are presented in square brackets “[]”. X represents any amino acid, while numbers in round brackets “()” indicate the number of residues allowed in this position.

1. Download the amino acid sequences from all the completed sequenced prokaryotic/eukaryotic genomes or individual species genome from the The National Center for Biotechnology Information (NCBI) ftp site (ftp://ftp.ncbi.nlm.nih.gov/).
2. Merge all of these sequences to produce a local database.
3. Download the full-length coding and amino acid sequences of 20 *CNGC* genes of *Arabidopsis thaliana* CNGC family from The Arabidopsis Information Resource (http://www.arabidopsis.org/), which are used as reference sequences for identification of homologs in other plants.
4. Using BlastP algorithm in Blast+ Program, the 20 reference AtCNGC proteins are used as first round database query sequences to search for homologs CNGCs in target plant genome by taking one AtCNGC protein at a time with a cutoff *E*-value < 1 × e−05. Alternatively, the reference AtCNGC sequences can be used as a query in different public sequence databases including TIGR (The Institute for Genomic Research, http://www.tigr.org/), PlantGDB (Plant Genome Data-base, http://www.plantgdb.org/), JGI (Joint Genome Institute, http://genome.jgi-psf.org/), NCBI ( http://blast.ncbi.nlm.nih.gov/Blast.cgi), Phytozome, ensemble, and/or specie-specific database such as MaizeGDB (Maize Genetics and Genomics Database, http://www.maizegdb.org/), BRAD (Brassica database, http://brassicadb.org/brad/), etc., using BlastP algorithm with a cutoff *E*-value of 10^−5^ or 0.
5. During Blast searches, retrieve only those sequences that show similarity >75% to the query sequence, with alignment over the stretch of 498 amino acids that is ∼70% of the length of CNGC of *A. thaliana*.
6. To avoid redundancy, select only one coding sequence from an organism’s *CNGC* gene by keeping the longest.
7. After first round, use the retrieved sequences as seeds to search against the local database with the same criteria described above.
8. Input the amino acid sequences of the retrieved candidate genes in the domain analyses programs [HMMER, Pfam, SMART, or CDD]. Sequences containing both cNMP-binding domain (IPR000595) and transmembrane/ion transport protein (PF00520) domain are recognized as CNGC proteins (Fig. 1a). Discard the truncated and irrelevant sequences from analysis.
9. The final step for plant CNGC identification is the presence “PBC” (Phosphate Binding Cassettes) and “hinge region with in the cNMP-binding domain”. To do this test:
  - Merge the amino acid sequences of CNGC family of the target specie in single FATSA file.
  - Perform multiple sequence alignment by MEGA (instructions given below) or Clustal W v2.0 program (http://www.ebi.ac.uk/Tools/clustalw2/).
  - Export alignment in FASTA format and view in GenDoc program.
  - Manually checked these aligned sequences for the presence of consensus motif key: “[L]-X(0,1,2)-[G]-X(3)-G-X(0,1,2)-[E]-L-[L]-X-[W]-X-[L]-X(7,37)-[S]-X(10,11)-[E]-[X]-[F]-X-[L]” at 90% conservation [24]. Amino acids allowed in a specific position are presented in square brackets “[]”. X represents any amino acid, while numbers in round brackets “()” indicate the number of residues allowed in this position.
  - The consensus motif key for hypothetical CNGC proteins is given and explained in Fig. 1b.

### Nomenclature and classification of plant CNGCs

**Figure 2:**
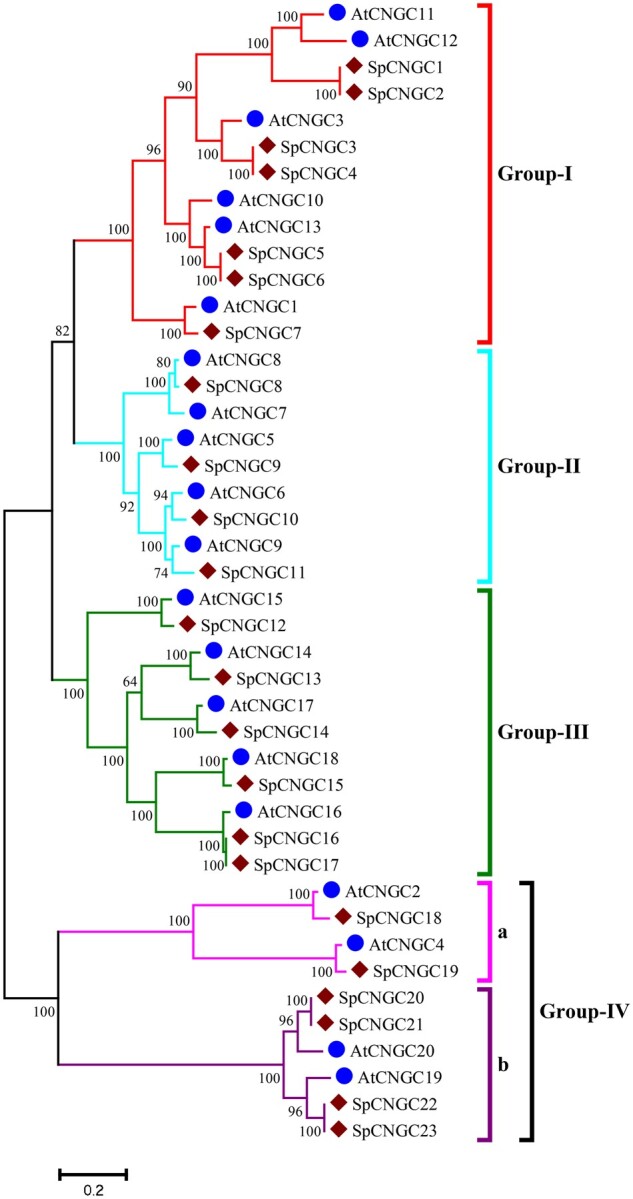
Exemplary phylogenetic tree showing the typical classification, nomenclature and relationship of CNGCs between target plant species (i.e. *Schrenkiella parvula*) and model *A. thaliana*.

Since *CNGC* is an established gene family, and while working on already annotated genomes, the researchers do not need to go through “International Protein Nomenclature Guidelines” for novel family. However, to avoid ambiguity in analyzing large data set containing multiple genes from different species and assess the evolutionary relationship between CNGC paralogs and with *Arabidopsis* orthologs, it is important to classify and assign a valid scientific name to identified member genes of an organism’s CNGC family. Among other, one of the standard methods for this phylogenetic analysis is to determine the relationship between a newly identified *CNGC* sequences to their characterized homologs (i.e. *A. thaliana* CNGCs). The stepwise method is described below:

1. Copy and paste the amino acid sequences of reference AtCNGC proteins and newly identified candidate CNGCs of the target specie and save in a single FASTA format file.
2. Download MEGA software for your operating system (https://www.megasoftware.net/) that supports sequence alignment using both the ClustalW and MUSCLE programs.
3. Open Alignment Explorer in MEGA, click create a new alignment, import the FASTA file, and select Alignment from the menu, then either ClustalW or Muscle.
4. Set the alignment parameters to the values you wish or leave the options alone to use the default parameters. Click Compute/OK.
5. The aligned sequences will replace the previously unaligned sequences in the Alignment Explorer. Export the alignment to MEGA or FASTA format for analysis.
6. Select “Phylogeny” from menu followed by maximum likelihood tree construction using Jones–Taylor–Thornton model with desired [No. of bootstrap replication = 1000; Gaps/Missing data treatment = Partial deletion] or default parameters. Click Compute/OK.
7. After the completion of process, the groupings of CNGC family are determined based on the classification of AtCNGCs: Group-I = AtCNGC1, AtCNGC3, and AtCNGC10- AtCNGC13; Group-II = AtCNGC5–AtCNGC9; Group-III = AtCNGC14–AtCNGC18; Group-Iva = AtCNGC2 and AtCNGC4; Group-IVb = AtCNGC19 and AtCNGC20.
8. Rename the newly identified *CNGC* genes either on the basis of their sequence homology to the reference AtCNGC homologs or from the beginning to the end of phylogenetic tree starting from *CNGC1* and so on.
9. Export the generated tree in desired format or save session for later use (Fig. 2).

### Phylogenetic analysis of plant CNGCs

**Figure 3:**
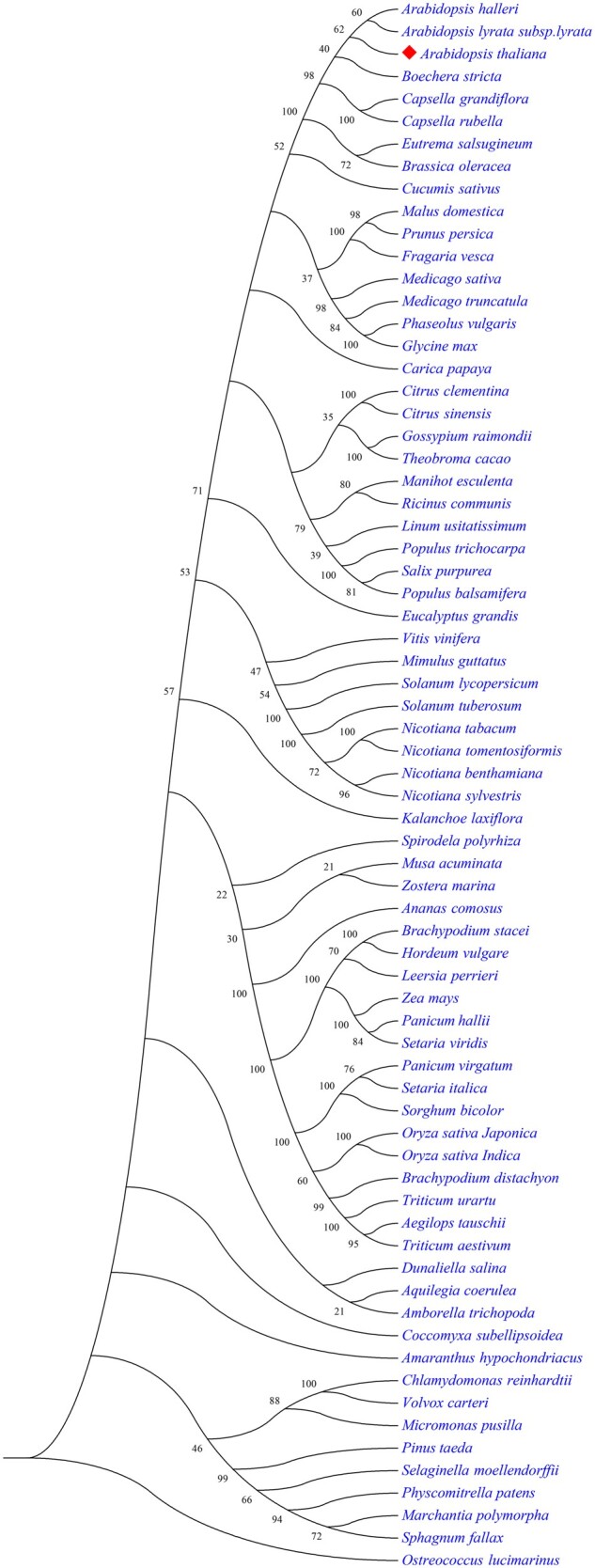
Exemplary tree showing the evolution of CNGC1 orthologue in all plant linage.

**Figure 4:**
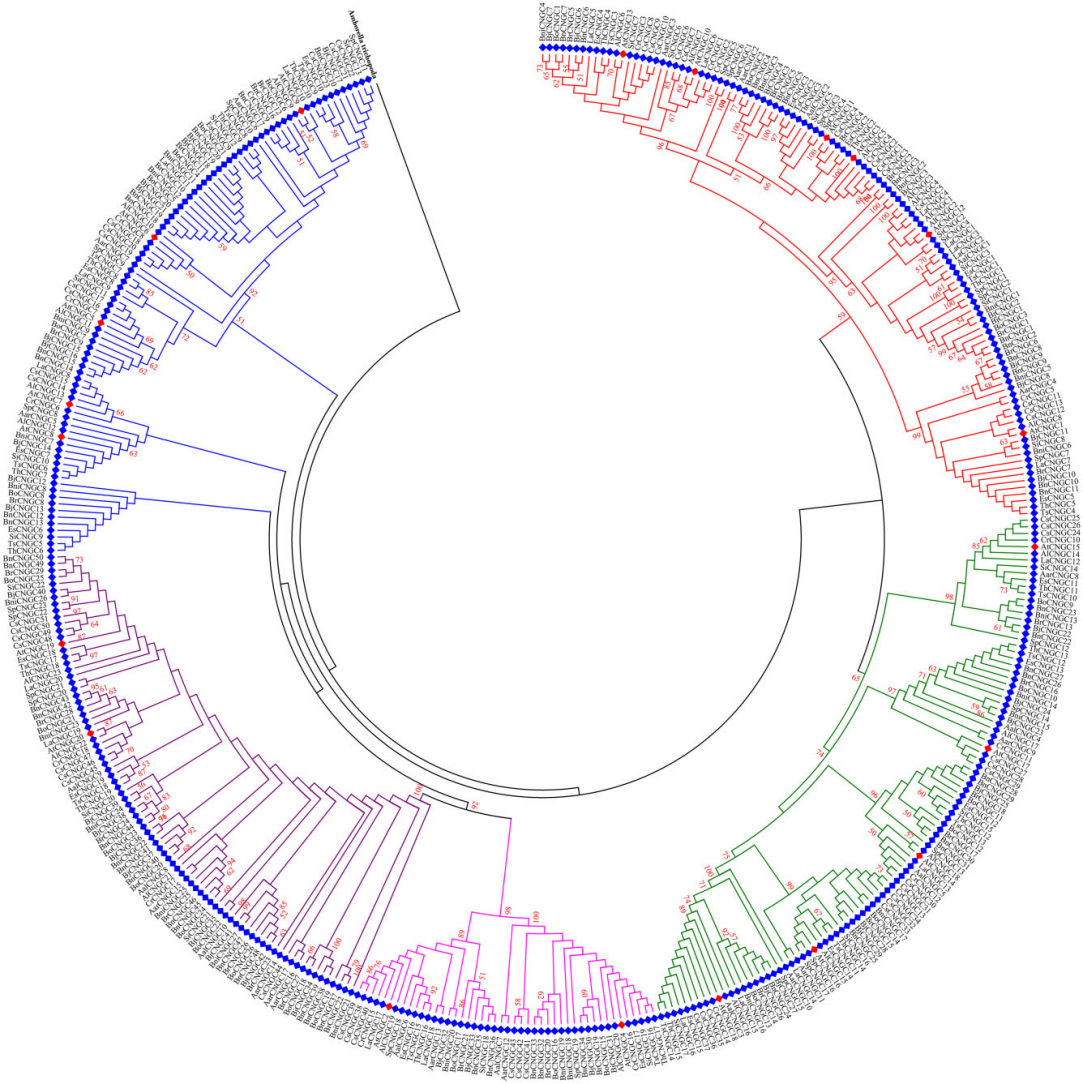
Example of rooted maximum likelihood tree showing the relationship of CNGCs between the species of Brassicaceae plant family using *Amborella trichocarpa* (Amborellaceae) as outgroup.

**Figure 5:**
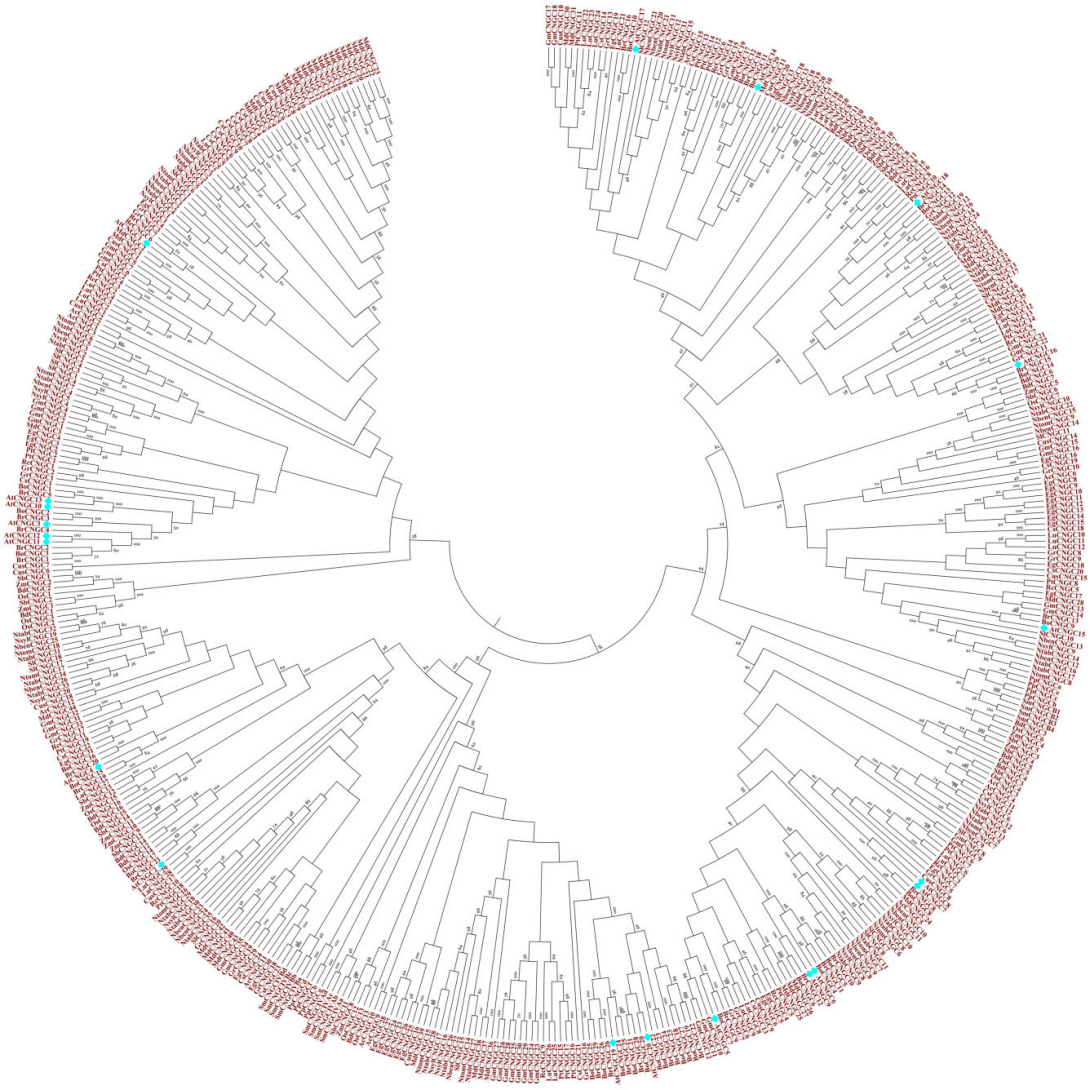
Exemplary phylogenetic tree showing the evolutionary relationship of CNGCs between all plant linages focusing on *A. thaliana* CNGCs. The analysis involved 513 amino acid sequences of *CNGC* genes from 24 plant species including target AtCNGCs marked with blue diamonds (Table 1). The evolutionary history was inferred by using the maximum likelihood method based on the Jones–Taylor–Thornton matrix-based model in MEGA 6.0. Bootstrap values of 1000 replicated are shown on each node.

To study the origin and evolution of *CNGC* gene family and explore the phylogenetic relationship among CNGC paralogs in plants, a comprehensive phylogenetic analysis is usually performed with (i) *CNGC* genes of two or more species (Fig. 2), (ii) single orthologs *CNGC* gene of different plant species (Fig. 3), CNGCs from particular plant group (Fig. 4), or all plant linages (Fig. 5 and Table 1). Generally, protein sequences are preferred in phylogenetic analysis due to the larger number of characters allowed in sequence string (20 amino acids compared to ATGC), sensitivity of amino acid blast search compared to DNA, conserved motifs/domains recognition, and lesser effects of synonymous codons on protein product.

**Table 1:** List and nomenclature of plant CNGC families used for phylogenetic analysis in current method

Species	Genes	Species	Genes
*Aquilegia coerulea*	*AcCNGCs*	*Nicotiana benthamiana*	*NbenCNGCs*
*Arabidopsis thaliana*	*AtCNGCs*	*Nicotiana sylvestris*	*NsylCNGCs*
*Brachypodium distachyon*	*BdCNGCs*	*Nicotiana tabacum*	*NtabCNGCs*
*Brassica oleracea*	*BoCNGCs*	*Nicotiana tomentosiformis*	*NtomCNGCs*
*Brassica rapa*	*BrCNGCs*	*Oryza sativa*	*OsCNGCs*
*Citrus sinensis*	*CsCNGCs*	*Physcomitrella patens*	*PpCNGCs*
*Cucumis sativus*	*CusCNGCs*	*Populus trichocarpa*	*PtCNGCs*
*Eucalyptus grandis*	*EgCNGCs*	*Ricinus communis*	*RcCNGCs*
*Glycine max*	*GmCNGCs*	*Selaginella moellendorffii*	*SmCNGCs*
*Gossypium raimondii*	*GrCNGCs*	*Solanum lycopersicum*	*SlCNGCs*
*Linum usitatissimum*	*LuCNGCs*	*Sorghum bicolor*	*SbCNGCs*
*Malus domestica*	*MdCNGCs*	*Zea mays*	*ZmCNGCs*

1. After naming as mentioned above, the amino acid sequences of the target CNGC genes and their orthologs CNGCs in plants are combined in single FASTA file.
2. Multiple sequence alignment is performed in MEGA or Clustal W v2.0 program (http://www.ebi.ac.uk/Tools/clustalw2/) with the default parameters.
3. The quality of alignment can have an enormous impact on the final phylogenetic tree [25, 26]. To exclude the poorly aligned positions, gaps, and divergent regions from the phylogenetic analyses, it is required to select only conserved blocks of the alignment using GBlocks 0.91b program [26, 27]. Alternatively, the amino acid sequences of conserved cNMP-binding domains of each *CNGC* gene of each family is collected and aligned via above cited programs.
4. Optional step: Predict the best-fit model for maximum likelihood (ML) optimizations and tree-building analyses by implementing the Akaike information criterion using ProtTest v1.4 [28] in PhyML program [29].
5. Construct a rooted maximum likelihood tree from Gblocks alignment/conserved cNMP-binding domains using MEGA, PhyML, or relevant programs under the Jones–Taylor–Thornton model. The sequence of the orthologous CNGC of *Chlamydomonas reinhardtii* can be used as an outgroup.
6. The reliability of interior branches is assessed with 1000 bootstrap resampling.
7. Additionally, construct three more phylogenetic trees with MEGA by using the neighbor joining, minimal evolution, and maximum parsimony methods, respectively.
8. Phylogenetic analysis produces Tree that can be can be displayed and edited in MEGA and Adobe illustrator, respectively. The tree diagram orders and connects the CNGC sequences reflecting homology and divergence between paralogs and ortholog CNGCs, and their genealogical relationship. The inner nodes of branch correspond to hypothetical common ancestors, while the branch lengths reflect the degree of diversification between two nodes. Moreover, researchers can observe if the *CNGC* genes of target plant species arose before or after different taxonomic clades such as monocots and dicots.

### Analysis of structural evolution of plant *CNGC* genes

To examine the structural evolution of CNGC genes family in terms of intron losses, intron gain which may have occurred during the structural evolution of CNGC paralogs, the structures of the individual CNGC family are determined as follows.

**Figure 6:**
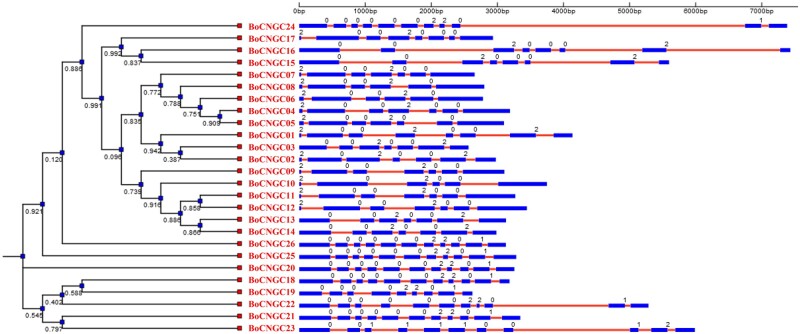
Diagram showing the exemplary output of the evolutionary analysis of plant *CNGC* gene structures. In this example, the gene structures reflecting exon–intron organizations and intron phases of 20 *Arabidopsis CNGC* genes. The NJ phylogenetic tree of CDs is shown on the left side of the figure, and the intron phases are shown with number [0, 1, and 2]. Phase-0-intron occur in between complete codons; phase-1-intron are separated by the first codon; phase-2 introns are located between the second and third nucleotides of a codon. The lengths of each exon and intron can be mapped to the scale given at the bottom.

1. Download the full-length nucleotide genomic and CDS sequences of CNGCs.
2. Copy and paste these sequences into separate files of genomic and CDS in FASTA format.
3. Replace the old IDs with new names assigned to each gene during phylogenetic analysis, then arrange the sequences in ascending order in each file and save as FASTA.
4. Open the website for Gene Structure Display Server (latest version), then choose sequence (FASTA) format in options.
5. Input both CDS and genomic sequences by importing FASTA files or directly pasting.
6. Depending on the type comparison/evolutionary analysis, upload a phylogenetic tree for inputted genes in NEWICK format. Click submit.
7. To further facilitate evolutionary analysis, user can include extra features by displaying intron phases and modifying the existing options after the generation of first figure.
8. The generated figure can be edited in built-in SVG-editor or in adobe illustrator after exporting as PDF.
9. Final results are concluded by comparing the intron number, intron length, intron positions, intron phases, and splicing sites among individual CNGC genes, phylogenetic groups, and plant linages to calculate the loss of exonic segments, acquisition of exonic segments, and conservation of exonic segments (Fig. 6).

### Molecular evolutionary analysis of plant CNGC proteins

The comparative analysis of amino acid composition depends upon the number of taxonomic groups and/or the number of CNGC proteins (single gene or whole family).

#### Method I

The method for small-scale study involving the comparison between different members/groups, or orthologs of two species is given below:

1. Copy and save the collected sequences into a single FASTA format file.
2. Separate FASTA file is made for the domain sequences of single CNGC family of an organism.
3. Each file is imported and aligned using Clustal X software or MEGA as mentioned above.
4. The alignment is saved in FASTA format.
5. Depending on the objective of study different types of evolutionary tests can be performed using the built-in tests in MEGA or following the instruction by Shckorbatov and Berezhnoy for manual analysis [23].

#### Method II

**Figure 7:**
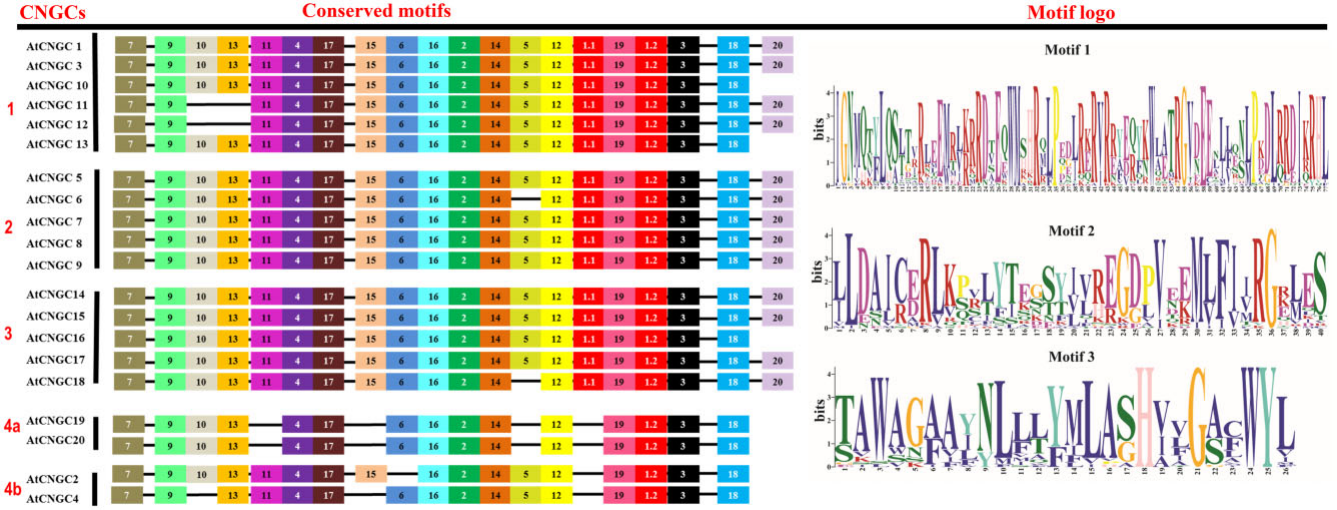
Diagram showing the exemplary output of conserved motifs and their logos studied during evolutionary analysis of plant CNGC proteins. In this example, 20 conserved motifs were identified in *Arabidopsis* CNGC proteins. Motifs are represented by numbers in colored boxes. Logos on the right reflects the conservation of amino acids in each motif, where the height of the individual amino acid shows the degree of conservation. The order of the motifs corresponds to the motif positions in the protein sequence. However, the length of the box does not correspond to the length of the motif.

For evolutionary analysis and comparison of CNGCs family between different taxonomic groups (genus, family, order, class, phylum) the following method is used:

1. Collect the sequences of a functional domain such as cNMP-binding domain or IQ domain from full-length amino acid sequences of CNGC proteins from each specie by using Pfam or SMART server (Fig. 7).
2. Save the file in FASTA format for each CNGC family of a selected specie or taxonomic group.
3. Perform multiple sequence alignment, export as FASTA file and view in GeneDoc program.
4. Deduce the consensus motif key spanning the PBC and hinge region within binding domain (CNBD) of each specie or taxonomic group using the method described by Zelman *et al*. [30], Nawaz *et al*. [16, 17], and Kakar *et al*. [15].
5. To evaluate the evolutionary pattern in terms of conservation or divergence of important amino acid residues within functional domains, the consensus keys can be compared between different taxonomic groups and to higher taxonomic rank.

#### Method III

**Figure 8:**
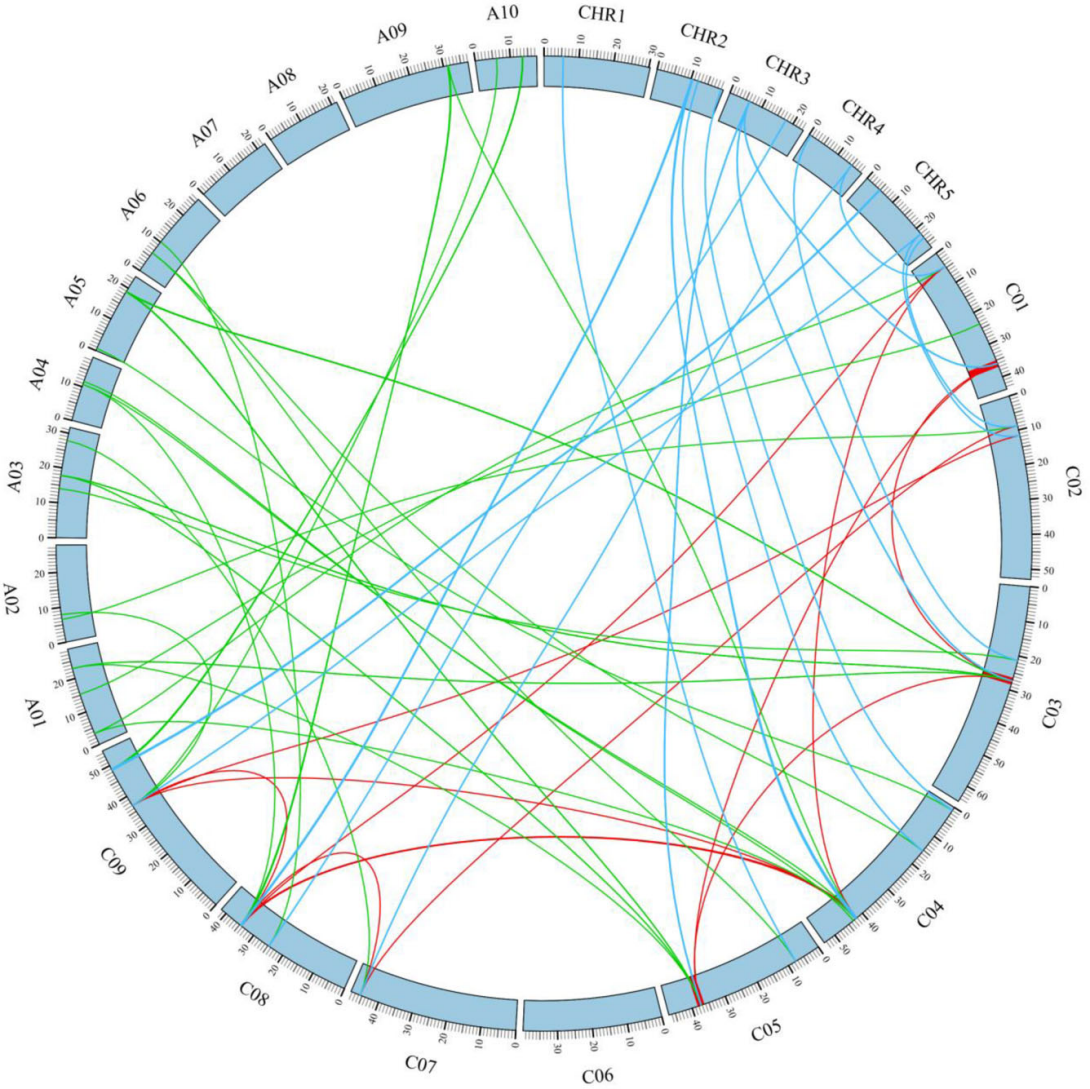
Sample circus plot showing the syntenic relationship of *CNGC* genes between the three plant species. Syntenic map shows highly conserved syntenic relationships based on orthologous pair positions of *CNGCs* between *B. rapa* (A01–A10), *A. thaliana* (Chr1–Chr5), and *B. oleracea*.

1. The FASTA format amino acid sequences of CNGC family of single or group of species are used as input in MEME suit, which can be downloaded or using the online portal http://meme-suite.org/.
2. The user-defined threshold options are set depending on the number of sequences and motifs. For CNGCs usually optimal motif width can be set between 6 and 200 with maximum number of different motifs as 10. Click submit.
3. The generated conserved motifs extracted motifs are annotated with domain/motif analysis programs.
4. The conserved MEME motifs and their sequence logos showing the degree of amino acid reside conservation are compared between paralogs and orthologs CNGCs (Fig. 8).
5. The output diagrams can be edited and subsequently displayed along with consensus tree or separately.
6. Additionally, the rates of molecular evolution of orthologs CNGC sequences from target plant species can be determined by applying codon evolution models to the aligned Open Reading Frames following the procedure described by Akhunov *et al*. [31].
7. The general physicochemical properties of CNGC proteins including molecular weights (kDa), aliphatic and instability indexes, ratio of charged residues, isoelectric points, and grand average of hydropathy calculated using the ProtParam tool (http://web.expasy.org/protparam/) and compared to support previous observations [32].

### Analysis of gene duplication events in plant CNGC evolution

Duplication events play important role in the expansion of plant gene family [33]. The following methods are used to investigate the occurrence of tandem and segmental duplication during the evolutionary analysis of plant CNGCs.

1. Perform multiple sequence alignment on amino acid sequences of CNGC proteins of selected plant species.
2. Construct a maximum parsimony phylogenetic from a complete alignment of CNGC proteins with bootstrap values from 1000 replicates indicated at each node.
3. Paralogs gene pair located at terminal nodes of phylogenetic tree showing high homology and overall identity of >50% can be considered as possible duplicates.
4. Obtain 10 protein-coding genes that are upstream and downstream of each pair of paralogs from genomic database.
5. Finally, the genes flanking one *CNGC* gene are matched to the genes flanking the other *CNGC* gene in the same pair. If these sequences reside within a region of conserved protein-coding genes, the paralogous *CNGC* gene pair is regarded as the result of a segmental duplication event.
6. Obtain the locus information (start and stop position) of *CNGC* genes from genomic database or using map-drawing programs for newly sequenced genome.
7. Tandem duplications are randomly defined as ones that occur within a sequence distance of 50 kb [34].
8. The *CNGC* gene accessions or proteins sequences can be used as input queries in Plant Genome Duplication Database (PGDD) to test if the *CNGC* gene pairs belong to conserved syntenic blocks or arose via segmental duplication.
9. The results of identified *CNGC* gene pairs/duplicates can be further validated via detailed syntenic analysis and comparison between gene structures, domain/motif compositions, and expression profiles.

### Syntenic analysis of plant *CNGC* genes

Some plant genomes have undergone through multiple whole-genome duplication events and their genomes are divided into sub-genomes [35]. For example, *Brassica rapa* and *B. oleracea* are ancient polyploids, whose genome have undergone whole-genome triplication event approximately 13–17 million years ago after divergence from *A. thaliana*, followed by large-scale chromosomal diploidization [36]. In such cases, syntenic gene analysis is very important for studying genome evolution and gene loss by comparing conserved flanking regions between two genomes.

1. To check collinearity between two genomes, protein-coding genes from different plant species are collected from public database such as Phytozome (v11).
2. An all-to-all alignment is performed by BLASTP with an *E*-value cut-off 1e−5 using the available alignment tool/program.
3. Then Multiple Collinearity Scan X (MCScanX) program is used to identify syntenic blocks between target plant species with the gap size ≤15, and syntenic genes ≥5.
4. Final diagram can draw using Circos plots (circos.ca).

### Synonymous and nonsynonymous substitutions

To further understand the evolutionary dynamics of plant CNGCs, the users can estimate the Ka (nonsynonymous substitution rate)/Ks (synonymous substitution rate), Ka and Ks ratio of duplicate gene pairs, or orthologs CNGCs of related plant species. The following analysis can be performed via MEGA or DnSP program on the basis of phylogenetic relationship between gene pairs (intra-family or inter-families) or gene duplicates, and/or in protein-coding/noncoding regions by using both exons and introns, or exons and flanking regions. For clarity, the users are advised to assign noncoding and coding protein regions to separate data files using standard protocols.

#### MEGA

1. The CDS/gene sequences of target *CNGC* gene pairs are aligned through ClustalW using MEGA.
2. Export alignment in MEGA format. Go to main menu of MEGA software and select “Compute Pairwise Distances”.
3. A new window will open, import the saved MEGA file and select the options given in Fig. 7.
4. The output will display a table containing Ka values if the users have chosen “Nonsynonymous sites” in the option, and Ks for “Synonymous sites”.
5. Click “average” from menu to get overall Ka or Ks value.
6. Repeat steps 2–5 to calculate Ka/Ks values as MEGA software calculate only Ka or Ks in single run and return the output table.

#### DnSP

1. Download and install the latest version of DnSP software on your system.
2. Perform alignment on CNGC CDS/Gene sequences using MEGA or clustalW. Save the alignment in FASTA/*. Meg or NEXUS format.
3. Open DnSP program and select “Open Data File” that will import the saved *CNGC* gene/CDS alignment file from desired location on computer’s drive.
4. Click the given options for confirming the properties of input data file. Click close.
5. Go to the main interface, and select “Synonymous and Nonsynonymous Substitutions” from analyses menu.
6. Click on the relevant option to define the desired region for analysis.
7. The output file will display the results in tabulated format:
8. Following conclusions can be drawn from Ka/Ks ratio:
  - Ka/Ks ratio = 1 implies neutral evolution (drift) showing that there have been equal number of synonymous and nonsynonymous substitutions between the ancestral and current version of CNGC proteins.
  - Ka/Ks ratio >1 indicates positive selection or adaptive evolution suggesting that there has been positive selection or evolutionary pressure to divert gene structure/function from ancestral state. This could lead to pseudogene formation, subfunctionalization, neofunctionalization, and subneofunctionalization.
  - Ka/Ks ratio <1 denotes negative selection implying that there has been evolutionary pressure to conserve the ancestral state of *CNGC* gene.
9. The positive selection/pressure over *CNGC* genes in target specie can be evaluated by performing multiple tests including: “McDonald and Kreitman test (MKT)” for Neutrality Index or determining in which sites the differences are fixed [37], CODEML and Phylogenetic Analysis by Maximum Likelihood to calculate the site-to-site ω variation [38, 39] using available protocols.

## Notes

1. Typical plant CNGC protein must contain an ion-transport or 1–6 transmembrane domains, CNBD with an overlapped calmodulin-binding domain, and/or IQ domain, respectively (Fig. 1a).
2. Naming starts with the first letter initials of genus and species, respectively (i.e. At for *A. thaliana*/Bo for *Brassica oleracea*) followed by CNGC and a number starting from 1. For example, AtCNGC1–AtCNGC20/BoCNGC1–BoCNGC26. In order to distinguish the two organisms having the same first letters of genus and species names, extra letters are added from specie name. For example, the correct naming of CNGCs from *Nicotiana tabacum* and *N. tomentosiformis* will be NtabCNGC and NtomCNGC rather than NtCNGCs. For further detail, refer to Nawaz *et al*. [17].

## Conclusion

The *CNGC* is an important gene family playing diverse biological functions in both plants and animals. In plant genomics research, performing genome-wide study of a gene family (e.g. *CNGCs*) provides valuable information such as the current status of gene family, their origin, expansion and evolution, structural and functional conservation, and divergence and studying complex regulatory mechanisms such as protein–protein interactions, cis-acting elements, miRNA targeting, and role in signaling pathways. Despite its importance, identification, characterization, origin, and evolution of CNGC family has not been well understood in many plants. This developed protocol enabled researchers to properly identify, characterize, and evolutionary study of the *CNGC* gene family in plants whose genomes are sequenced and publicly available. Therefore, the consequences of the current study will undoubtedly provide a foundation and drive the research forward to the next level, where the researchers can select and clone novel candidate *CNGC* genes to study signaling pathway mechanisms in detail and make newly improved cultivars through molecular breeding.

## Author contribution

K.U.K. and A.A.B. designed and conceptualized this study. The identification and characterization were performed by M.M., A.A., S.U.K., and A.L. Evolutionary portion was performed by A.A.B. along A.B. A.A.B. and S.U.K. wrote the article along K.U.K. All authors commented at each stage. KUK supervised the study. All the authors have read and agreed to the published version of the article.

## Funding

This research did not receive any specific grant from funding agencies in the public, commercial, or not-for-profit sectors.

## Conflict of interest

 The authors declare no competing interests.

## Data availabilty

The datasets were derived from sources in the public domain: The Arabidopsis Information Resource database at http://www.arabidopsis.org/; The National Center for Biotechnology Information (NCBI) database at ftp://ftp.ncbi.nlm.nih.gov/; Brassica database (BRAD) at http://brassicadb.cn/.
